# Interoception and symptom reporting: disentangling accuracy and bias

**DOI:** 10.3389/fpsyg.2015.00732

**Published:** 2015-06-04

**Authors:** Sibylle Petersen, Ken Van Staeyen, Claus Vögele, Andreas von Leupoldt, Omer Van den Bergh

**Affiliations:** ^1^Research Group on Health Psychology, KU LeuvenLeuven, Belgium; ^2^Institute for Health and Behaviour, University of LuxembourgLuxembourg, Luxembourg

**Keywords:** classification, interoception, bias, accuracy, decision strategies, sensitivity, negative affect, symptom report

## Abstract

Anxiety and anxiety sensitivity are positively related to accuracy in the perception of bodily sensations. At the same time, research consistently reports that these traits are positively related to bias, resulting in the report of more and more intense symptoms that poorly correspond with physiological dysfunction. The aim of this study was to test the relationship of accuracy and bias in interoception. Furthermore, we tested the impact of individual differences in negative affect and symptom report in daily life on interoceptive accuracy and bias. Individuals higher in symptom report in daily life and negative affect were marginally more accurate in an interoceptive classification task in which participants were asked to identify different respiratory stimuli (inducing breathing effort) as belonging to a high or low intensity category. At the same time, bias in overestimating intensity of stimuli was significantly increased in participants higher in symptom report and negative affect, but only for more ambiguous stimuli. Results illustrate that interoceptive accuracy and bias need to be considered independently to understand their interaction with psychological factors and to disentangle (mis)perception of bodily sensations from liberal or conservative perceptual decision strategies.

## Introduction

Nothing is closer to us than our own body but few things seem so elusive than the perception of bodily sensations. The brain is not passively ‘measuring’ signals from the body, but these signals interact with emotions and influence decision-making, behavior, and attention which in turn can change (perception of) bodily signals (Damasio, 1994; Craig, 2004). It is thus little surprising that correlations between self-reported bodily sensations and physiological changes are usually low in healthy individuals and in patients with somatic disease (e.g., Banzett et al., 2000; Petersen et al., 2011). Research consistently reports that negative affect, anxiety, and anxiety sensitivity are related to stronger nocebo effects (Van den Bergh et al., 1997, 2004) and higher levels of symptom report (Watson and Pennebaker, 1989; Petersen et al., 2011). Furthermore, in anxiety disorders, elevated self-report of somatic sensations is combined with a tendency for catastrophizing interpretations (e.g., Barlow, 1988; Barsky et al., 1994; Carleton et al., 2014).

In contrast to these low correlations between self-report and physiology particularly in individuals high in negative affect, a meta-analysis reports medium to strong effect sizes for a positive relationship between accuracy in heartbeat detection and psychological variables such as anxiety sensitivity and anxiety (Domschke et al., 2010). Higher anxiety sensitivity is also related to lower detection thresholds for respiratory stimuli (Petersen and Ritz, 2011). Furthermore, a study measuring respiratory-related evoked potentials (RREPs) in participants watching negative emotional pictures found increased later components of RREPs in individuals higher compared to lower in anxiety (von Leupoldt et al., 2011). The authors interpreted these increases as higher motivated attention toward respiratory signals in more anxious individuals in negative affective contexts.

Thus, while individuals higher in negative affective states and traits seem to be more biased in interoception, they seem at the same time to be more accurate. Attempts to reconcile these seemingly paradox findings are faced with a methodological challenge. Research on interoceptive accuracy uses mostly heartbeat detection tasks (e.g., Domschke et al., 2010). In one type of heartbeat detection task, participants are asked to decide whether a sound signal is matching their heartbeat (e.g., Asmundson et al., 1993; Harver et al., 1993). Data collected in such a task could be used to differentiate bias and accuracy in a signal detection approach. Unfortunately, performance levels are around chance in the majority of participants, raising questions about the usefulness of this paradigm (Jones, 1994; Domschke et al., 2010). Most studies on interoception use another type of heartbeat detection task and follow a mental tracking paradigm in which participants are instructed to count their heartbeats during intervals of different length (Schandry, 1981). This paradigm, however, cannot differentiate sensitivity and bias.

Confounding sensitivity and bias means to lose crucial information. At the same level of sensitivity, individuals can apply liberal or conservative decision strategies which may result in very different forms of coping behavior following either a liberal “better safe than sorry” or a conservative “wait and see” approach (Macmillan and Creelman, 2004; Lynn and Barrett, 2014).

Making perceptual decisions and classifying bodily sensations, for example, as symptom or as benign sensation, is an inherent part of interoception (Petersen et al., 2014) and may present a challenge (Carleton et al., 2014). A headache may be painful, but *perhaps* not painful enough to take medication; breathlessness may be strong, but *perhaps* not strong enough to signal that we should stop exercise; heartbeat may be elevated, but *probably* not a sign of a heart disease. Although these sensations are clearly above detection threshold they are ambiguous regarding their category. We cannot be 100% sure about classification of ambiguous sensations at the border of two interoceptive categories, but we can optimize decision strategies by weighting the risk of missing a symptom against the risk of false alarms, that is, the risk of classifying a benign sensation as pathological (Griffiths et al., 2008). These processes of forming probabilistic beliefs about bodily sensations have been suggested to underlie interoception at every stage of information processing and may not always be deliberate or even consciously accessible (Edwards et al., 2012).

In this study, we tested the relationship between accuracy and bias in an interoceptive classification task in which participants were asked to correctly categorize different respiratory stimuli (respiratory loads increasing breathing effort) as belonging to either a low or a high intensity category and to indicate their place within these categories by labeling them as A1, A2, A3, A4, B1, B2, B3, and B4, with increasing numbers being related to increasing stimulus intensity (Petersen et al., 2014). A novel feature of this study is that we tested classification of a number of stimuli varying in intensity and assigned to two categories. Most signal detection paradigms test only one (usually low intensity) stimulus representing the signal against signal absent trials (for exceptions, see Kepecs et al., 2008; Yang et al., 2014). Interoceptive categories, however, usually subsume a range of different signals (e.g., signs of airway obstruction in asthma may come in different degrees of intensity and a variety of symptoms on multiple dimensions may fall in the category ‘cold symptoms’) and an important task is to classify ambiguous signals as belonging to one of various sensation classes.

We tested accuracy in the classification of multiple stimuli as belonging to one of two intensity categories. Furthermore, we tested how bias changes across the different stimuli within interoceptive categories. We expected that it would be more challenging to classify sensations closer to the border of two interoceptive categories than for more prototypical/central sensations, for example, a stimulus which is not clearly high or low, but moderate. It is important to note that also if non-categorization information would be given, an A1 stimulus would be more easily classified as low than an A4 stimulus. We tested whether interoceptive bias would be higher for more ambiguous sensations and whether individual differences in negative affect and symptom report in daily life would affect this bias. Higher increase in bias toward the shared category border could be interpreted a strategy to rather risk false alarms than to miss signals which indicated potential harm (Lynn and Barrett, 2014). Lower increase in bias toward the category border could be interpreted as the category border being incorporated into interoceptive decision-making, serving as a red flag and reducing misclassification particularly for stimuli at this category border.

We expected that higher bias for more ambiguous stimuli (better safe than sorry approach ignoring the category border) would lead to more accurate results in the classification task (as predicted by Lynn and Barrett, 2014), explaining the seeming paradox of higher bias and higher accuracy in individuals with negative affective expectations toward symptoms or high in general negative affect.

## Materials and Methods

### Participants

Participants were 54 women (mean age 21.04 years, SD = 1.8) without known chronic or acute disease. They were selected based on prescreens for high and low habitual report of bodily symptoms in daily life using the Checklist for Symptoms in Daily Life (Wientjes and Grossman, 1994). Participants completed this symptom checklist consisting of 39 bodily sensations (e.g., tingling in arms and hands, back pain, etc.) with regard to how often they had experienced these sensations within the last year (scale 1 *never* -5 *very often*). The questionnaire has good reliability (Chronbach’s alpha 0.70–0.90, Wientjes and Grossman, 1994). The pre-screen was filled in by 355 individuals and mean score was 91.95 (SD = 18.59). We invited participants with values in the symptom checklist of either higher than 80 or lower than 60 consecutively from this prescreen list.

Participants completed the same checklist also after the laboratory appointment. After the task, four participants from the high symptom reporter group did no longer reach high values on the symptom checklist and were excluded from the analysis. Thirteen of the participants in the low symptom report group reached values of 61–75 on the symptom checklist after the task (which induced symptoms), and were included in the low symptom report group. This resulted in *n* = 25 for the low symptom report group and *n* = 25 for the high symptom report group. Participants signed informed consent prior to participation. They received course credit or reimbursement for participation. The study was approved by the local ethics committee.

### Instruments

We induced feelings of breathing effort by presenting eight different respiratory loads using the instrument Powerbreathe K5 (POWERbreathe International Ltd., Southam, UK). The instrument allows gradually increasing breathing resistance at inhalation by reducing the diameter of the breathing port. We used the software Breathelink to program presentation of loads. Exhalation was unrestricted. Resistances of the eight respiratory loads were chosen to ensure that differences between adjacent breathing loads could be distinguished by healthy volunteers, but were similar enough to leave an uncertainty margin for classification (7, 9, 11, 14, 18, 23, 28, and 37 cmH_2_O).

Participants completed the Checklist for Symptoms in Daily Life (Wientjes and Grossman, 1994) twice during the experimental session. Firstly, they were asked to indicate which of the symptoms they had perceived in the last year to test whether results of the pre-screen were reliable. Secondly, we asked them to indicate which symptoms they had experienced during the actual task. We used the Positive and Negative Affect Schedule (Watson et al., 1988), a five point rating scale assessing negative and positive affect within the last 4 weeks with ten mood related adjectives as items for each subscale.

### Protocol

At the start of the protocol, participants signed an informed consent form giving information about the study. In the first block of the interoceptive classification task, we presented the eight loads in random order, each load four times for two breaths. During presentation, the label of the load was presented on a computer screen (label A1 together with the 6 cmH_2_O load, A2 – 9 cmH_2_O, A3 – 11 cmH_2_O, A4 – 14 cmH_2_O, label B1 – 18 cmH_2_O, B2 – 23 cmH_2_O, B3 – 28 cmH_2_O, and B4 – 37 cmH_2_O). Participants were instructed that in the following block, they would have to solve a categorization task. They were asked to memorize the sensation and the label so that they would be able in a second block, when loads were presented without label, to indicate the correct category (A or B) and the location of the stimulus within its category (1–4). In previous studies, we found that participants are able to distinguish and label eight different loads assigned to two categories after this training procedure above chance level (Petersen et al., 2014). Furthermore, in a study comparing a group of participants receiving category information and a control group receiving the information that stimuli were labeled with numbers only (increasing consecutively from lowest to highest stimulus), we found that categorization and an arbitrary category boundary indeed change perception of interoceptive stimuli. Differentiation between stimuli falling into the same categories was reduced and discrimination between categories was more pronounced in the experimental compared to the control group (Petersen et al., 2014, Study 1). Similar results were found in studies on visual perception using arbitrary category boundaries in a similar paradigm (Tajfel and Wilkes, 1963; Corneille et al., 2002), and in a study using an odor classification paradigm where mice were trained to classify six odor stimuli as belonging to one of two similar odor categories (Kepecs et al., 2008).

In Block 2, loads were presented again in pseudo-randomized order with 18 presentations per load, that is, 144 load presentations overall, with two breaths per load presentation. We presented the loads in a way that each load was preceded at least once by every other load to reduce the impact of order effects. Participants were asked to classify each load by assigning the correct labels (A1–B4). They did not receive feedback on their performance. After Block 2, participants completed the PANAS, the symptom checklist for symptoms last year, and the symptom checklist for symptoms directly after the task. Finally, we asked them to answer demographic questions on age, height, weight, and chronic or acute disease.

### Data Analysis

We used SPSS 20 for all analyses and used the SPSS syntax for *c* and *d’* indices proposed by Stanislaw and Todorov (1999). We calculated *d’_class_* measures reflecting how accurate participants were in distinguishing categories A and B. In contrast to signal detection, classification is a choice between two types of signals and not between signal and noise. Still, the formula is identical, only that A or B is treated as ‘noise’ and the other category as ‘signal.’ We calculated one mean *d’_class_* index as mean of a *d’_Aclass_* (treating A as signal and B as noise) and *d’_Bclass_* (treating B as signal and A as noise).

Furthermore, we used signal detection analysis to calculate classification criteria *c_class_*. We calculated *c_class_* = -(z(H) + z(F))/2, with z(H) as z-transformed hit rates of rating A if a load of Category A was presented, and z(F) as z-transformed false alarm rates of reporting A1 if a load from B was presented. This resulted in four *c*_class_ indices per category (*c*_classA1_, *c*_classA2,_ *c*_classA3,_ *c*_classA4_, *c*_classB1_, *c*_classB2_, *c*_classB3_, and *c*_classB4)_. Thus, *c_class_* reflects bias, taking into account classification errors and correct classification with c indices below zero indicating a liberal bias and c indices above zero indicating a conservative bias.

In a classification paradigm, the meaning of the terms liberal and conservative depends on the standard used. A tendency to require little evidence to classify a stimulus as B is liberal in a “better safe than sorry” approach, if missing B is regarded to be more costly than missing A. We calculated *c_class_* indices in a way that the term ‘liberal’ refers to a decision strategy that favors classifying A as B for stimuli actually belonging to A, or classifying B as A for stimuli actually belonging to B. In other words, a liberal tendency in our analysis is a strategy that favors misclassification. We expect this liberal tendency to be strongest for more ambiguous stimuli and to become weaker for clearly high or clearly low stimuli.

We tested in a univariate analysis of covariance (ANCOVA) whether *d’_class_* would differ between groups of high and low habitual symptom reporters, controlling for negative affect and symptoms experienced during the task as covariates. In further repeated-measures ANCOVAs we tested whether *c_class_* values would be significantly reduced as functions of closeness to the shared category border between A and B. The within-individual factor was Classification with four levels of the within-individual factor Classification per category (*c*_classA1_, *c*_classA2,_ *c*_classA3,_ *c*_classA4_/*c*_classB1_, *c*_classB2_, *c*_classB3_, *c*_classB4_). We included Symptom Report (high/low) as between-individual variable to test whether individuals higher in habitual symptom report would show more liberal tendencies to assign stimuli to the B category. Again, we included negative affect and symptoms experienced during the task within the ANCOVA model as covariates.

As alternative way of analysis, we performed an individual regression slopes analysis, following the procedure described by Pfister et al. (2013). In this analysis, we tested whether the individual regression slopes of participants indicating a decline toward a more liberal bias from A1 to A4 and an increase to more conservative bias from B1 to B4 would be steeper for participants in the High Symptom Report group. We compared the single slopes in a one way ANCOVA with negative affect as covariate and symptoms reported directly after the task included as control variable. Furthermore, we calculated the absolute value for the slopes across Categories A and B and compared, whether steepness of slopes would differ between A and B in a repeated-measures ANCOVA with negative affect as covariate, controlling for symptoms experienced during the task. Furthermore, in the supplemental material we present data on accuracy of differentiation within categories.

## Results

**Table 1** summarizes mean values across groups of high and low symptom reporters for self-report scales. We tested group differences in a *t*-test for independent groups.

**Table 1:** Participants’ characteristics (standard deviation in parentheses).

	Symptom experienced last year	Negative affect	Symptoms experienced during the task
Low symptom reporters	60.12^∗^ (SD = 7.05, range: 42–75)	16.84^∗^ (SD = 7.10, range: 10–34)	57.04^∗^ (SD = 9.24, range: 41–75)
High symptom reporters	99.88 (SD = 14.06, range: 81–138)	24.48 (SD = 7.84, range: 11–28)	78.68 (SD = 16.47, range: 41–107)

*^∗^p < 0.001.*

### Accuracy of Classification

**FIGURE 1:**
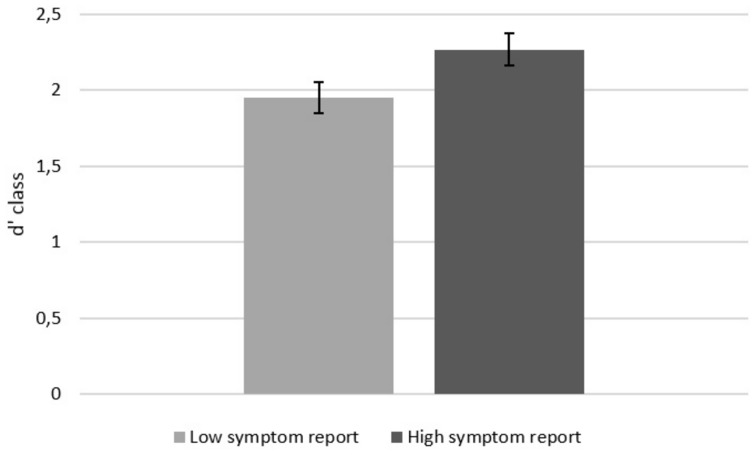
**Accuracy in classifying loads as belonging to A or B**. Error bars represent standard errors of the mean.

High symptom reporters were marginally more accurate classifying loads as A or B, main effect for Symptom Report *F*(1,49) = 3.60, *p* = 0.064, $\eta_{p}^{2}$ = 0.073 (**Figure 1**). Effects for negative affect and symptoms reported during the task were not significant, *F*s < 1.

### Misclassifications and Response Bias

**FIGURE 2:**
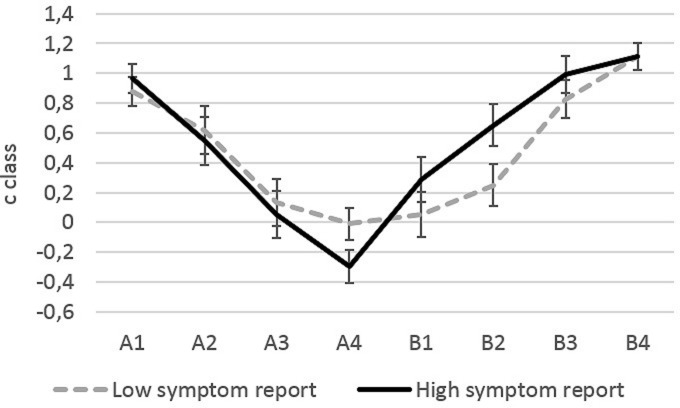
***C*_class_ values calculated from z-transformed correct identification rates of a load as A or B and z-transformed false alarm rates**. Error bars represent standard error of the mean.

Classification bias *c_class_* per load became less conservative toward the shared category border between A and B in both groups, but this decrease was significant only for Category A *F*(3,44) = 18.27, *p* < 0.001, = 0.555, and not for Category B *F*(3,44) = 2.06, *p* = 0.119, $\eta_{p}^{2}$ = 0.123 (**Figure 2**, statistics are reported with the covariate Negative Affect). Please note that the lack of significant results for Category B does not necessarily imply that the effect for Category A was larger than for Category B. In the next section, we report a comparison of effects with individual slope analysis. The decrease in Category A, was significantly stronger in high symptom reporters, interaction Classification X Symptom Report *F*(3,44) = 3.97, *p* = 0.014, $\eta_{p}^{2}$ = 0.213. This interaction was non-significant for category B, *F*(3,44) = 1.65, *p* = 0.192, $\eta_{p}^{2}$ = 0.101. In other words, across Category A, participants high in habitual symptom report showed a more pronounced change from a conservative to a liberal criterion than participants low in habitual symptom report. Also after exclusion of the covariate Negative Affect from this model, the interaction effect of Classification X Symptom Report remained significant, *F*(3,44) = 3.18, *p* = 0.033, $\eta_{p}^{2}$ = 0.175.

For Category A, the interaction of the within-individual factor Classification with the covariate Negative Affect was marginally significant, *F*(3,44) = 2.29, *p* = 0.092, $\eta_{p}^{2}$ = 0.135, but not for Category B, *F*(3,46) = 2.06, *p* = 0.120, $\eta_{p}^{2}$ = 0.123. None of the other effects was significant (all *F*s < 1.18).

*Post hoc* tests revealed that none of the single *c_class_* indices differed significantly between groups regardless whether negative affect was included in the model or not (all *p*s > 0.103). We performed explorative *post hoc* tests with *t*-tests for independent groups to test whether the *c_class_* index for A4 indicated indeed a *liberal* bias or only a *less conservative* bias. This explorative *t*-test testing differences from zero, suggests that only for high habitual symptom reporters, *c_class_* for A4 was significantly smaller than zero, that is, only in this group bias changed significantly from conservative to liberal *t*(24) = -2.327, *p* = 0.027.

### Individual Slopes Analysis

Individual slopes analysis revealed that the shift toward a liberal bias (as expressed in a negative slope for Category A) was stronger in the High Symptom Report group (mean: -0.386, SD = 0.143) compared to the Low Symptom Report group (mean: -0.357, SD = 0.126), *F*(1,47) = 4.59, *p* = 0.037, $\eta_{p}^{2}$ = 0.089. The effect of negative affect was non-significant and we report statistics without including negative affect as covariate. For Category B, the shift to a more conservative bias from B1 to B4 did not differ significantly between groups of Low (mean: 0.366, SD = 0.159) and High Symptom Report (mean: 0.293, SD = 0.184), *F*(1,47) = 2.34, *p* = 0.128, $\eta_{p}^{2}$ = 0.049. Comparing the absolute value of single slopes (ignoring the direction of slope) in a repeated-measures ANCOVA revealed that the steepness of slope was marginally higher for Category A (mean: 0.375, SD = 0.125) than for Category B (mean: 0.339, SD = 0.155), main within-individual effect *F*(1,46) = 3.27, *p* = 0.077, $\eta_{p}^{2}$ = 0.065. The interaction with the between-individual factor Symptom Report was significant, *F*(1,47) = 4.64, *p* = 0.036, $\eta_{p}^{2}$ = 0.090. While for participants from the Low Symptom Report group the absolute amount of slopes for Category A (mean: 0.357, SD = 0.126) and B (mean: 0.366, SD = 0.158) were not significantly different (*p* = 0.828), the absolute amount of slopes differed significantly for the High Symptom Report group between Category A (mean: 0.392, SD = 0.125) and B (mean: 0.311, SD = 0.150) with a steeper slope for Category A. In other words, change in bias across categories was stronger for category A than for Category B, but only in the High Symptom Report group. Again negative affect had no significant effect and including it did not change patterns of significance and we report results without including this covariate.

### Correlations between Bias and Accuracy

**FIGURE 3:**
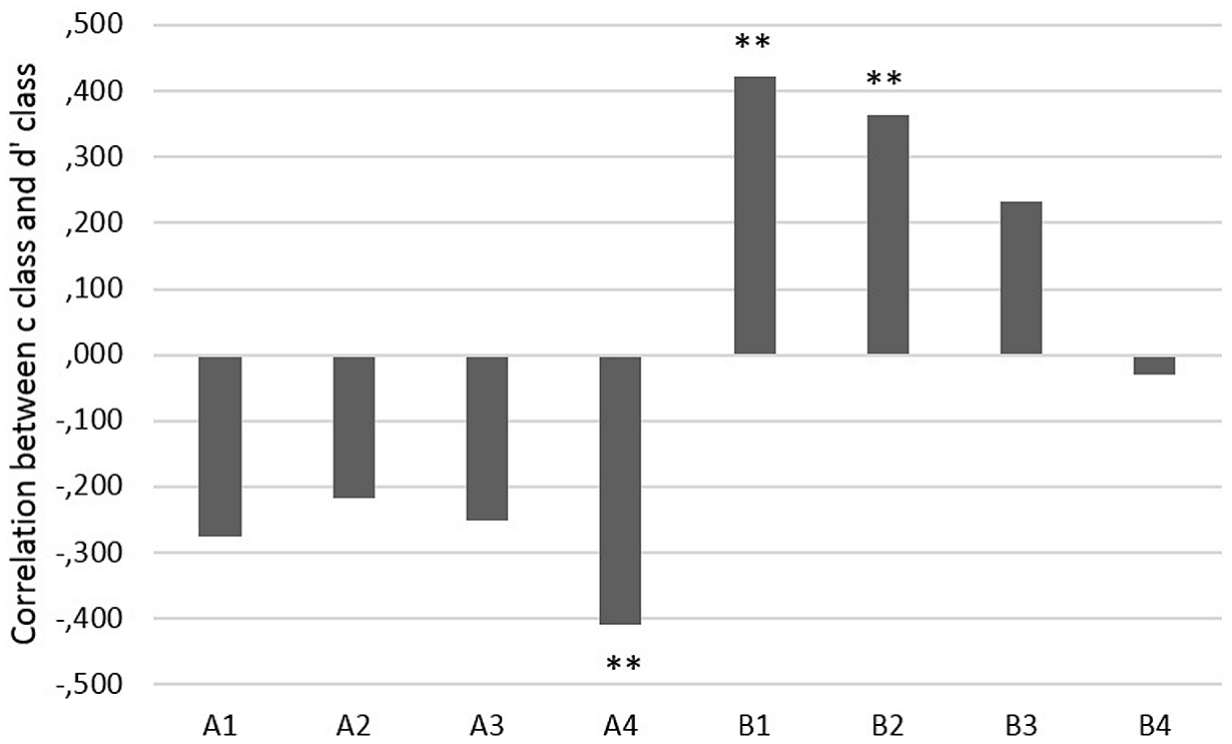
**Correlation coefficients between *c_class_* indices and *d’_class_* (^∗∗^*p* < 0.01)**.

Correlations (Pearson’s correlation coefficient, two-tailed, **Figure 3**) between *d’_class_* and *c_class_* were negative for category A, but significant for stimulus A4 only. In other words, higher accuracy was related to a more liberal decision criterion for the stimulus at the category border of A. This ‘better safe than sorry’ approach for this particularly ambiguous stimulus was related to overall better classification results. Furthermore, *d’_class_* was significantly positively related to *c_class_* for stimulus B1 and B2, that is, higher accuracy was related to a more conservative decision criterion (being reluctant to decide that stimuli B1 and B2 belonged to A) for stimuli at the border of category B. None of the other correlations was significant, *p*> 0.053.

## Discussion

Participants higher in symptom report in daily life scored higher on negative affect and reported more symptoms during the experimental task. They were also marginally more accurate in the interoceptive classification task. This is consistent with results of prior research showing a relationship between interoceptive accuracy/awareness and negative affective traits and states (e.g., Domschke et al., 2010; Petersen et al., 2011; von Leupoldt et al., 2011). A novel finding of this study is that classification strategies changed across categories. Bias became more liberal with increasing closeness to the border between categories. This change from conservative to more liberal decision strategies was significantly stronger in high symptom reporters and positively related to classification sensitivity *d’_class_*. Furthermore, for Category A (but not for B), the decline of individual slopes was steeper in the High compared to the Low Symptom Report group. Thus, the information that there was a category border between A4 and B1 was effectively reducing bias toward the category border, but only in participants who did not have strong affective expectations about stimuli.

Results confirm hypotheses proposed recently in signal detection research. Lynn and Barrett (2014) have suggested that increased uncertainty about visual stimuli will lead to more extreme decision strategies in signal detection tasks. These more extreme strategies for more ambiguous stimuli are suggested to *optimize* decision-making and behavior (Lynn et al., 2012). Lynn and Barrett (2014) give the example of a person who walks more carefully in a dimly lit room compared to a brightly lit room (i.e., under higher or lower ambiguity of visual signals). Optimizing speed relative to sight allows avoiding injury or breaking objects. They suggest that “extreme bias may reflect not an impairment, but a normal adaptive mechanism that offsets the single impairment, poor sensitivity” (Lynn and Barrett, 2014, p. 1670). Calibrating bias to increased uncertainty for ambiguous sensations (closer to category borders) may be highly adaptive. More extreme decision strategies under higher ambiguity (closeness to category borders) were more successful in the present study. Higher classification accuracy (*d’*_class_) was related to *more* bias at the borders of categories and increased bias in this laboratory task was related to bias in symptom report in daily life.

It is important to note that the border of the two neutrally labeled categories A and B was not intrinsically meaningful, as it would be the case for a border that marks a transition between a sensation and a symptom category (e.g., increased airway resistance which is either benign or indicates an oncoming asthma attack). It could be questioned whether arbitrary category boundaries as such affected perception. Prior research using a design with an experimental group receiving category information and a control group for which stimuli were numbered consecutively from lowest to highest without categories has found that arbitrary category boundaries between categories A and B affect interoception (Petersen et al., 2014, Study 1). Differentiation between categories was increased compared to differentiation within categories in the experimental group (receiving category information) compared to differentiation between identical stimuli in control groups (not receiving category information). Similar effects have also been found for visual perception (e.g., Tajfel and Wilkes, 1963; Corneille et al., 2002). Furthermore, a study testing classification decisions in mice which were trained to classify six odor stimuli as belonging to two categories (which shared a boundary and for which similarity between stimulus A3 and B1 was the same as between A3 and A2) found that detection of higher ambiguity for stimuli closer to category boundaries does not require meta-cognition and that ambiguity determines speed of decisions, overall accuracy, and is correlated with prefrontal cortex activity (Kepecs et al., 2008). For meaningful category labels and borders, or a paradigm which would trigger meta-cognition in participants about the costs of misclassifying stimuli, results might be even stronger than observed in this minimal paradigm using neutral labels.

It is tempting to speculate that the observed increasingly liberal bias for ambiguous stimuli at category borders in high symptom reporters may be a first step toward interoceptive threat-generalization. If a stimulus at a category border is consistently misclassified as belonging to a more intense category, this may lead in the long run to establishing a new and lower category border. This process may continue until more and more stimuli, which were initially in a low (or “safe sensation”) category, are subsumed in a higher (or “dangerous symptom”) category. Following this lead, fear-generalization in anxiety disorders (Lissek et al., 2008) may be interpreted as increasingly more liberal strategies spreading from category borders to more and more stimuli within a category.

Ambiguity of sensations because of their location close to a category border and their no longer clearly low or clearly high magnitude may be clinically as relevant as ambiguity related to a poor signal to noise ratio of sensations (i.e., minimal stimulation such as heartbeat at rest). Research on the role of uncertainty in panic disorder found that intolerance of uncertainty was substantially and significantly related to symptom report in panic disorder even after controlling for anxiety sensitivity (Carleton et al., 2014). Results from this correlation study suggest that patients seemed to find uncertainty about the decision whether a clearly detectable sensation is a sign of pathology (or not) at least as aversive as the sensation as such. Probing deeper into interoceptive classification strategies in anxiety disorder, future research should test whether the relationship of negative affective states and traits and interoceptive accuracy and bias is mediated by increased feelings of aversiveness related to uncertainty about classification of sensations as pathological or benign. Trait constructs such as intolerance for ambiguity which are closely related to anxiety may be interesting in that regard (Birrell et al., 2011).

Negative affect was increased in individuals high in habitual symptom report. Negative affect has been suggested to be related to a general lack of inhibition and not specifically to interoception (Bogaerts et al., 2015), but again, our results suggest that this effect is not a general effect across all types of stimuli within a category, but higher for more ambiguous stimuli. The relationship between negative affect and perceptual bias has been confirmed in a large number of studies (e.g., Bar-Haim et al., 2007; Cisler and Koster, 2010), but testing decision strategies under uncertainty may help to shed light on the processes underlying this relationship.

A limitation for generalization of the present results is that categories were distinct and defined only by one dimension (inspiratory resistance). Interoceptive categories are multidimensional and may overlap on some dimensions and be distinct on others. Sensations experienced during an asthma attack, for example, and sensations related to panic attacks belong to two distinct diagnostic categories, but are partly overlapping in how they are experienced by patients (Lehrer et al., 2002). Multidimensionality and overlap of categories in interoception may increase ambiguity of sensations, which in turn may lead to more extreme forms of bias for ambiguous sensations.

A further important limitation of this study is that we included only young female participants. Attention to interoceptive stimuli as well as expression of negative affect and fear may be higher in women than in men. Future research needs to address gender and age differences in interoceptive classification.

## Conclusion

The relationship between sensitivity and bias is not uniform across interoceptive categories. To understand the relationship between interoception and individual difference variables, paradigms are needed which do not confound sensitivity and bias and vary the degree of ambiguity stimuli have regarding their classification.

## Author Contributions

SP and OVdB contributed equally to the development of the study concept and study design. AvL contributed conceptually to the study design and data analysis. Testing, data collection, and data analysis were performed by SP, and KvS in collaboration with OVdB as senior researcher. All authors, SP, OVdB, KvS, AvL, and CV drafted the paper. All authors approved the final version of the paper for submission.

## Conflict of Interest Statement

The authors declare that the research was conducted in the absence of any commercial or financial relationships that could be construed as a potential conflict of interest.

## Supplementary material

The Supplementary Material for this article can be found online at: http://journal.frontiersin.org/article/10.3389/fpsyg.2015.00732/abstract
