# Supplementary material for: Interoception and symptom reporting: disentangling accuracy and bias
Source: Front Psychol. 2015 Jun 4;6:732. doi: 10.3389/fpsyg.2015.00732 (PMC4454884; doi:10.3389/fpsyg.2015.00732)
Supplement: Supplementary file 1 [file Presentation_1.PDF]

Supplemental material

Individual differences in anxiety are related to reduced differentiation between loads within compared to between categories (Petersen et al., submitted). Such categorization effects of reduced perceived variability within, but not between categories have been interpreted as (over-)generalization of category-related information across stimuli within categories (e.g., Corneille et al., 2002). Overgeneralizations and reduced attention to finer differences between sensations may also play a role in symptom over-report and catastrophizing about symptoms. In chronic pain, discrimination of pain-related bodily sensations is often impaired (Flor, Schugens, & Birnbaumer, 1992) and training of perceptual discrimination can reduce pain (Moseley, Zalucki, & Wiech, 2008).

We tested whether also individual differences in habitual symptom report in daily life would reduce perception of variability within categories in comparison to variability between categories. We calculated d' values within and between categories. These d' values reflect the likelihood of correct differentiation between neighbouring loads relative to the likelihood of confusing these loads (Macmillan & Creelman, 2004). For this purpose, we calculated mean d' values for sensitivity for differences between neighbouring loads within Category A (d' A1 and A2, d' A2 and A3, and d' A3 and A4) and Category B (d' B1 and B2, d' B2 and B3, and d' B3 and B4). Furthermore, we calculated sensitivity for differences between loads at the shared category border (d' A4 and B1). Please note that this d' for differentiation at the shared category border is not the same as d' for differentiating between A and B in general, for which analyses are reported in the main report about this study. Thus, we tested a repeated-measures ANOVA model with the three levels of the within-individual factor Categorization average d' between neighbouring loads within A, d' A4/B2, and average d' between neighbouring loads within B.

We tested, d' values within categories would be lower relative to d' values for differentiating between loads at the shared category border, that is, we tested the significance

of the quadratic effect for the within-individual variable Categorization (for a similar procedure, see Petersen et al., 2014). Furthermore, we tested whether this effect would be stronger in individuals high compared to low in habitual symptom report. We used SPSS 20 for all analyses and the SPSS syntax for *d'* indices proposed by Stanislaw and Todorov (1999).

Breathing behavior is an important factor in differentiation between loads. Just like pushing faster with a hand against a weight will result in higher perceived effort, applying higher flow rates (breathing in more liter air per second) will result in higher perceived breathing effort. Flow rates are often increased in individuals high in fear of suffocation which may magnify the experience of minor breathing restrictions and contribute to the sensation of breathing impairment (Alius et al., 2013; Pappens et al., 2012). Less is known on whether flow rates affect the ability to differentiate between loads.

Results

Differentiation between neighboring loads within Category A was close to chance and less accurate compared to differentiation between loads at the shared category border or within Category B, main effect of the within-individual variable Categorization $F(2,46)=14.43$, $p<.001$, $\eta_p^2=.380$ (Figure S1). The quadratic effect was marginally significant, indicating more correct differentiation between neighboring loads at the shared category border (between A4 and B1) compared to differentiation between neighboring loads within categories, $F(1,47)=3.33$, $p=.074$, $\eta_p^2=.065$. Post hoc tests for the groups of participants high and low in habitual symptom report showed a significant quadratic effect only for the group high in symptom report, $F(1,23)=5.15$, $p=.033$, $\eta_p^2=.177$, but not for the group low in symptom report, $F(1,23)=0.39$, $p=.537$, $\eta_p^2=.016$.

Participants higher and lower in symptom report in daily life did not differ significantly in flow rates (low symptom report: mean flow rates = 1.10, *SD*=.49; high symptom report: mean flow rates = .97, *SD*=.29). Flow rates interacted significantly with the

within-individual variable Categorization, but only for the group of high symptom reporters, $F(2,22)=4.017$, $p=.033$, $\eta_p^2=.268$, not for the group of low symptom reporters, $F(2,22)=1.68$, $p=.209$, $\eta_p^2=.133$. The quadratic effect was significant for high symptom reporters, $F(1,23)=6.89$, $p=.015$, $\eta_p^2=.230$, but not for low symptom reporters, $F(1,23)=2.14$, $p=.157$, $\eta_p^2=.085$, indicating that only in high symptom reporters, differentiation between neighboring loads within categories was significantly less accurate than between loads at the shared category border. This quadratic effect interacted significantly with mean flow rates for participants high in symptom report, $F(1,23)=4.36$, $p=.048$, $\eta_p^2=.159$, and only marginally significant for participants low in symptom report, $F(1,23)=3.51$, $p=.074$, $\eta_p^2=.132$. To explore the relationship of flow rates and categorization effects further, we calculated a mean categorization index by subtracting the average of the d’ values for A and B from the d’ value for differentiating between loads at the category borders (A4 and B1). We correlated this mean categorization effect index with flow rates (Pearson, two tailed). This correlation was negative, indicating that lower flow rates were related to higher categorization effects in participants higher in symptom report in daily life, $r(25)=-.399$, $p=.048$.

Discussion

As in prior research (Petersen et al., 2014), we found lower differentiation between neighboring loads within relative to between categories, but this effect was significant only in participants high in habitual symptom report. This is in line with research which found that anxiety is related to a decrease in differentiation within compared to between categories (Petersen et al., submitted). This suggests that higher expectations to experience symptoms in daily life are related to reduced attention to finer differences between sensations and to higher focus on the distinction between sensation categories. Interestingly, individuals high in habitual symptom report are not only (marginally) more successful in differentiating between categories if fairly easy distinctions such as between A2 and B2 are included (see main report for this study), but are also better in telling apart highly similar loads at the shared category

border (A4 and B1) relative to differences of comparable magnitude within categories. This suggests that in participants higher in symptom report in daily life, an arbitrarily placed category border (differences between categories in cmH_2O were not larger than differences within categories) affected interoception and induced generalization between stimuli within relative to between categories.

In contrast to prior research (Alius et al., 2013), we did not find significant differences in flow rate between participants, but this may have been due to the short presentation time of loads since effects of fear of suffocation and anxiety sensitivity on flow rates increase over time and are stronger after longer exposure to loads (Pappens et al., 2012). Despite the lack of group differences in flow rates, only in the group high in habitual symptom report, flow rates interacted significantly with the within-individual effect and the quadratic effect for Categorization. Lower flow rates were related to higher categorization effects in this group. This suggests that cognitively mediated effects of generalization within (artificial) interoceptive categories may be stronger in individuals with higher expectations to experience symptoms in daily life, but only if physiological factors such as higher flow rates do not reduce sensitivity for differences between loads. To test this hypothesis further, however, studies are needed which manipulate flow rates experimentally.

References

Alius, M. G., Pané-Farré, C. A., Von Leupoldt, A., & Hamm, A. O. (2013). Induction of dyspnea evokes increased anxiety and maladaptive breathing in individuals with high anxiety sensitivity and suffocation fear. *Psychophysiology, 50, 488*-497.

Corneille, O., Klein, O., Lambert, S., & Judd, C. M. (2002). On the role of familiarity with units of measurement in categorical accentuation: Tajfel and Wilkes (1963) revisited and replicated. Psychol Science 13, 380-383

Flor, H., Schugens, M. M., & Birbaumer, N. (1992). Discrimination of muscle tension in chronic pain patients and healthy controls. Biofeedback Self Regul, 17, 165-177.

Moseley, G. L., Zalucki, N. M., & Wiech, K. (2008). Tactile discrimination, but not tactile stimulation alone, reduces chronic limb pain. Pain, 137, 600-608

Macmillan, N. A., & Creelman, C. D. (2004). Detection theory: A user's guide. Psychology press.

Pappens, M., Smets, E., Bergh, O., & Diest, I. (2012). Fear of suffocation alters respiration during obstructed breathing. Psychophysiology, 49, 829-832.

Petersen, S., Schroijen, M., Moelders, C., Zenker, S., & Van den Bergh, O. (2014). Interoceptive categorization: the role of perceptual organization on processing sensations from inside. Psychol Science, 25, 1059-1066.

Petersen, S., Vögele, C., & Van den Bergh, O. (submitted). The Procrustes Effect: How anxiety modifies the perception of bodily sensations.

Stanislaw, H., & Todorov, N. (1999). Calculation of signal detection theory measures. Behav. Res Methods Instrum Comput, 31, 137-149.
