# Supplementary figures and images for: Interoception and symptom reporting: disentangling accuracy and bias
Source: Front Psychol. 2015 Jun 4;6:732. doi: 10.3389/fpsyg.2015.00732 (PMC4454884; doi:10.3389/fpsyg.2015.00732)

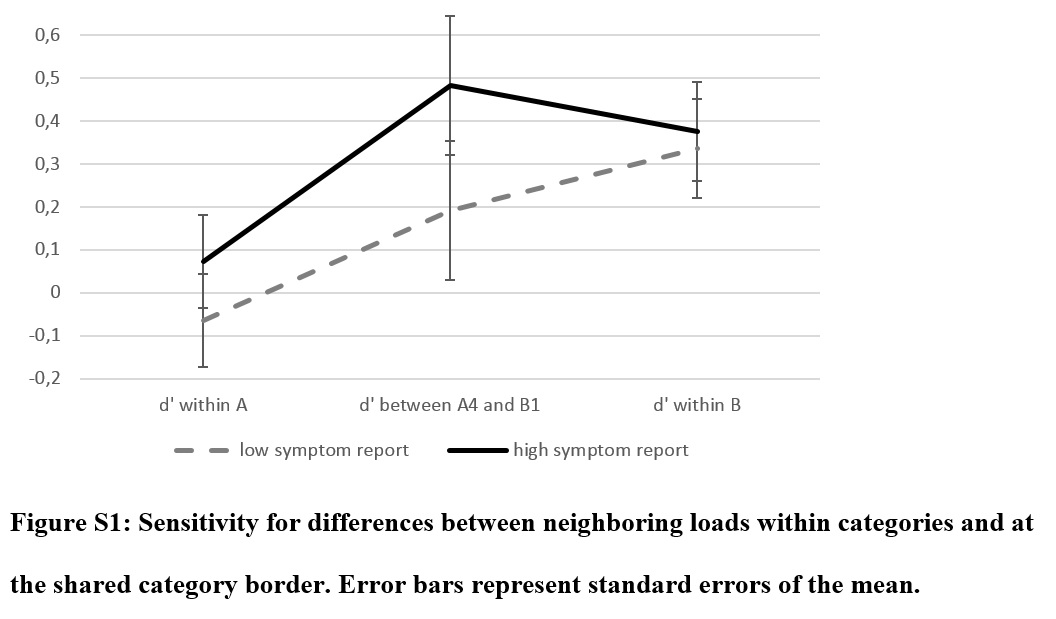

Supplement: Supplementary file 2 [file Image_1.JPEG]
